# Putting the “Sensory” Into Sensorimotor Control: The Role of Sensorimotor Integration in Goal-Directed Hand Movements After Stroke

**DOI:** 10.3389/fnint.2019.00016

**Published:** 2019-05-22

**Authors:** Lauren L. Edwards, Erin M. King, Cathrin M. Buetefisch, Michael R. Borich

**Affiliations:** ^1^Neuroscience Graduate Program, Graduate Division of Biological and Biomedical Sciences, Emory University, Atlanta, GA, United States; ^2^Department of Rehabilitation Medicine, Laney Graduate School, Emory University, Atlanta, GA, United States; ^3^Department of Neurology, Emory University, Atlanta, GA, United States; ^4^Department of Radiology and Imaging Sciences, School of Medicine, Emory University, Atlanta, GA, United States

**Keywords:** sensorimotor integration, motor learning, motor control, stroke, sensation

## Abstract

Integration of sensory and motor information is one-step, among others, that underlies the successful production of goal-directed hand movements necessary for interacting with our environment. Disruption of sensorimotor integration is prevalent in many neurologic disorders, including stroke. In most stroke survivors, persistent paresis of the hand reduces function and overall quality of life. Current rehabilitative methods are based on neuroplastic principles to promote motor learning that focuses on regaining motor function lost due to paresis, but the sensory contributions to motor control and learning are often overlooked and currently understudied. There is a need to evaluate and understand the contribution of both sensory and motor function in the rehabilitation of skilled hand movements after stroke. Here, we will highlight the importance of integration of sensory and motor information to produce skilled hand movements in healthy individuals and individuals after stroke. We will then discuss how compromised sensorimotor integration influences relearning of skilled hand movements after stroke. Finally, we will propose an approach to target sensorimotor integration through manipulation of sensory input and motor output that may have therapeutic implications.

## Introduction

Goal-directed movements of the hand are required to perform most tasks of daily living, such as tying a shoe, buttoning a shirt, and typing, among others. These highly coordinated voluntary movements involve interacting with and manipulating objects in the environment and rely on sensorimotor integration. Sensorimotor integration is the ability to incorporate sensory inputs that provide information about one’s body and the external environment to inform and shape motor output (Wolpert et al., 1998). More specifically, sensory inputs for goal-directed hand movements provide information in an egocentric reference frame detailing location, size, weight, and shape of an object. In addition, kinematic information about the hand and upper extremity, including the trajectory needed to interact with the object, is provided. Successful integration of information contributes to generating the most efficient motor plan to execute a given task. Additionally, ongoing sensory feedback during motor performance refines the motor plan to optimize current and future performance. This process of sensorimotor integration is often disrupted in neurological disorders, such as stroke.

Stroke is defined as infarction of central nervous system tissue attributable to ischemia, based on neuropathological, neuroimaging, and/or clinical evidence of permanent injury (Sacco et al., 2013). Stroke is the fourth leading cause of death and remains the number one leading cause of long-term adult disability (Benjamin et al., 2017). Furthermore, the loss of productivity after stroke currently costs the United States an average of $33.9 billion per year and is expected to reach $56 billion by 2030 (Ovbiagele et al., 2013), making stroke a public health crisis. A primary contributor to persistent disability after stroke is incomplete motor recovery (Lai et al., 2002). Spontaneous biological recovery of motor function occurs during the first months after stroke (Cramer, 2008), underlying a current emphasis on intensive early intervention, although results are often mixed and complex (Bernhardt et al., 2017a). Despite intensive therapy, upper extremity impairment resolves up to 70% of baseline function for a given patient with some patients showing even less recovery than predicted (Winters et al., 2015). Most stroke survivors are left with a limited ability to perform skilled hand movements necessary for daily functioning (Lang et al., 2013). To reduce disability after stroke, there is a need to improve our understanding of the neuronal network physiology necessary to regain skilled functional hand use.

**Figure 1:**
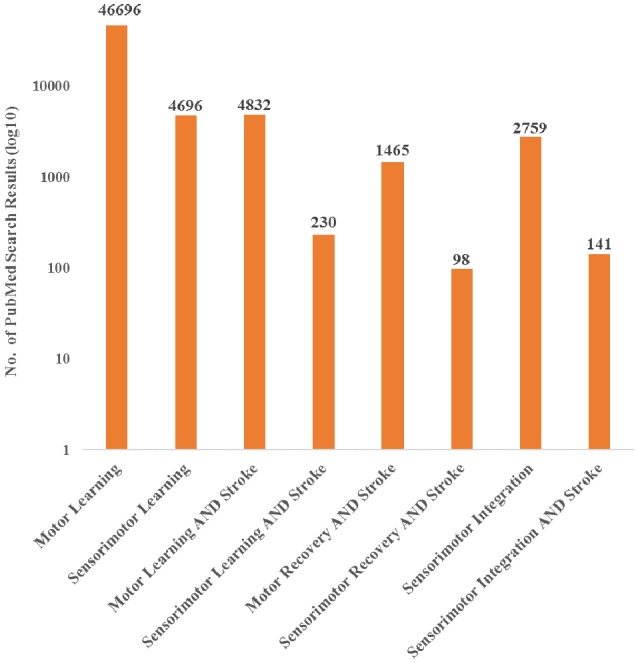
PubMed search results for both motor and sensorimotor aspects of learning and stroke recovery. More publications focused on motor learning and recovery than on both motor and sensory components of learning and recovery. Furthermore, there were a relatively small number of publications involving sensorimotor integration and stroke compared to sensorimotor integration overall. “Sensorimotor,” “Sensori-motor,” and “Sensory motor” were all used to ensure differences in terminology did not affect the search results. Additionally, “Sensory motor” and “Sensory-motor” produced the same search results.

Currently, the field has primarily investigated motor deficits and motor learning with limited consideration of the role of sensory information, even though it is recognized that integration of sensory information is a critical component of motor control (Borich et al., 2015; Bolognini et al., 2016). Furthermore, evidence has shown that sensory input is important for recovery after stroke. In a systematic review, Meyer et al. found that across six studies, the extent of deficits in proprioception and light touch of the arm and hand were significantly related to recovery after stroke (Meyer et al., 2014). Despite evidence that sensory input is a critical component to motor execution, research nomenclature has been primarily focused on motor characteristics post-stroke and has therefore not capitalized fully on the information a sensorimotor perspective could provide. This observation is supported by a literature search showing an emphasis towards motor recovery and learning after stroke, over sensorimotor recovery and learning, with limited focus on sensorimotor integration (Figure 1). While it is possible that authors may use these terms interchangeably, the literature search terminology suggests that there is potential bias towards motor contributions. Therefore, there is an important gap in our understanding of the contributions of sensorimotor integration to recovery.

In the following brief review, we will highlight the importance of processing and integrating sensory and motor information that underlies skill performance and learning with an emphasis on skilled hand movements in stroke. We will focus primarily on three cortical regions: primary motor cortex (M1), posterior parietal cortex (PPC) and primary somatosensory cortex (S1) while briefly mentioning other cortical and subcortical brain areas also involved in sensorimotor integration. These brain regions are highlighted due to our focus on the integration of sensory and motor information at the level of the cortex, but also because these cortical areas receive blood supply from the middle cerebral artery (MCA), which is the most common type of stroke (Walcott et al., 2014). Furthermore, all three brain regions contribute to the corticospinal tract (CST) that provide necessary contributions to executing and controlling skilled hand movements routinely used in daily life. It should be noted that strokes occur in other brain regions but usually have less of an impact on sensorimotor integration underlying goal-directed, skilled hand movements and are outside the primary scope of this review article.

In the first section of this review article, we will discuss the role of sensorimotor integration *via* M1, PPC, and S1 in normal, skilled hand movements. We will then discuss how sensorimotor integration is affected by stroke and how impaired sensorimotor integration can impact relearning of skilled hand movements. Last, we propose an approach to target sensorimotor integration by manipulating sensory input and restricting motor output that may have therapeutic implications for stroke recovery.

## The Role of M1 in Goal-Directed Hand Movements

### M1 Involvement in Movement Execution

The M1 has a critical role in the execution of voluntary movements. Upper extremity movement execution is particularly dependent on descending output from M1 through the spinal cord to upper limb muscles. Pyramidal neurons in layer 5 have axons that are bundled together as a significant portion of the CST, where 85%–90% of the fibers decussate in the pyramids to provide control to the hand contralateral to the hemisphere of the M1 (Rosenzweig et al., 2009). The remaining fibers, approximately 10%–15%, maintain ipsilateral projections that have a minor role in distal extremity motor control (Zaaimi et al., 2012). Of the neurons terminating in the spinal cord, some neurons will indirectly influence movements by synapsing onto interneurons in the intermediate zone (Rathelot and Strick, 2009) whereas direct control arises from the cortico-motoneuronal (CM) cells that terminate monosynaptically on α-motoneurons in the ventral horn of the spinal cord (Lemon et al., 1986). These α-motoneurons innervate skeletal muscle to control contralateral muscle contractions, and subsequently, voluntary movements (Rathelot and Strick, 2009; Schieber, 2011). The most abundant projections from M1 are to motor neurons that innervate hand muscles allowing for direct and individualized control of fingers required for complex and skilled hand movements (Dum and Strick, 1996). A lesion to these CST axonal fibers is the leading cause of motor disability and specifically causes loss in individualized finger function (Lawrence and Kuypers, 1968; Lemon, 2008), reiterating the importance of this connection from M1 to the α-motoneurons innervating muscles of the hand. While CST is the largest contributor to skilled hand movement, there are other pathways, such as the reticulospinal tract, that offer additional contributions to certain aspects of hand function (for review, see Baker, 2011). The topographical organization of M1 demonstrates a larger spatial representation for the hand reflecting the relative importance of the output from CM cells to the hand (Penfield and Boldrey, 1937). The populations of CM cells in M1 fire differentially to allow for a variety of functional uses of the hand (Griffin et al., 2015). Within these populations, individual neurons can be tuned to preferentially code for single or multiple fingers or more proximal joints (Kirsch et al., 2014), and the kinematics of a movement, such as direction, force, and speed are also encoded (Georgopoulos et al., 1982, 1992; Mahan and Georgopoulos, 2013). This level of specification in M1 neuronal tuning allows for the execution of an extensive repertoire of complex hand movements.

As mentioned previously, the execution of skilled hand movements by M1 requires sensory information. Representations of the external environment must be generated from visual, proprioceptive, and tactile input (Makino et al., 2016), and these representations are combined with internal representations of the motor system, such as hand position, to create an internal model (Blakemore et al., 1998). Both external and internal representations have inherent variability that can be reduced by incorporating input from multiple sensory modalities (Körding and Wolpert, 2004).

Successful multisensory integration contributes to execution of a motor command that results in the desired movement outcome. For instance, if the goal is to button a shirt, the internal model should include the position of the button and buttonhole and starting position of the hand. These positions are determined by visual, proprioceptive, and tactile information that will be processed through PPC [visual (Kaas et al., 2011)] and S1 [proprioceptive, tactile (Kim et al., 2015), and nociceptive (Liang et al., 2011)], Sensory information associated with manipulation of the button will also be provided. The relevant sensory information is then relayed to M1, where a motor command is generated. This internal model will also be influenced by prior motor execution that contributes to development of an efference copy of the motor output (von Holst and Mittelstaedt, 1950). Using this information, an internal model includes predictions about expected sensory feedback resulting from the generated movement (Flanagan et al., 2006). In this example, if the button is not at the correct angle required for it to go through the button hole, or if the hand is in the incorrect starting position, the sensory reafferent information occurring in response to movement will not align with the predicted feedback generated from the efference copy (von Holst and Mittelstaedt, 1950). Therefore, the predicted sensory consequence will be updated, the model adapted, and subsequently, the error will be corrected by adjusting the motor command (Shadmehr et al., 2010).

**Figure 2:**
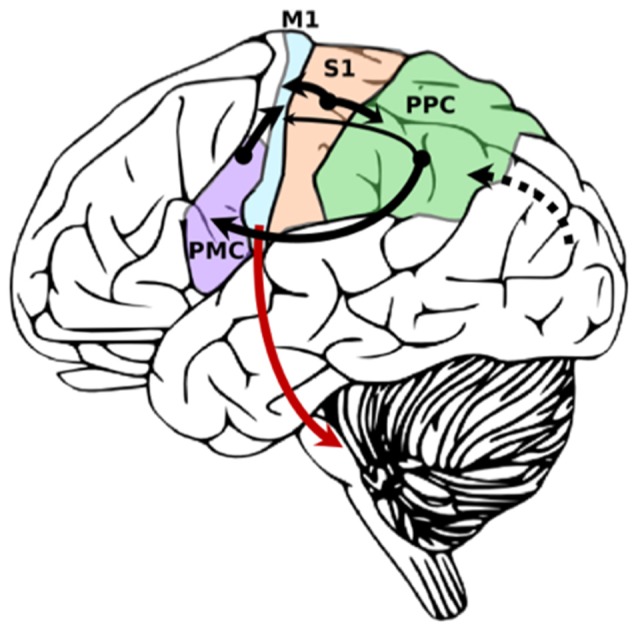
Simplified diagram demonstrating primary sensory inputs to primary motor cortex. Cortico-cortical connections are black. Cortico-fugal projections from M1 are red. Width of arrow denotes strength of connection. Dotted line denotes primary visual input from visual cortex into posterior parietal cortex (PPC) for multimodal integration.

There are several brain regions involved in sensorimotor integration for goal-directed hand movements (Figure 2). Non-cortical structures contributing to sensorimotor integration include the: basal ganglia (Nagy et al., 2006), cerebellum (Proville et al., 2014), and thalamus (Mo and Sherman, 2019). In rodents and primates, it has been shown that distinct subdivisions of the thalamus receive input from the basal ganglia and cerebellar nuclei and project to M1 (Bosch-Bouju et al., 2013; Bopp et al., 2017). The ventroanterior and ventromedial nuclei receive information from the basal ganglia, typically through GABAergic projections. The ventrolateral nucleus receives glutamatergic projections from cerebellar nuclei. In addition to these motor thalamic regions, there has been evidence from rodent models to suggest that sensory thalamic regions, such as the posterior medial nucleus, project directly to M1 (Ohno et al., 2012; Hooks et al., 2013, 2015). However, it is unclear whether these specific pathways are present in humans and non-human primates.

Here, our main focus is on sensory signals from PPC and S1 that convey pertinent information about somatosensation, proprioception, and visuomotor transformations to M1. The ability to transform visual and proprioceptive information about the location and space of the internal and external world is important to inform motor commands (Burnod et al., 1992). M1 neurons fire in response to both visual and proprioceptive stimuli (for review, see Hatsopoulos and Suminski, 2011). The M1 hand area is separated into caudal (M1c) and rostral (M1r) subregions: CM cells primarily arise from M1c and provide direct control of movements of the hand and distal forearm, whereas neurons in M1r influence motor control indirectly using interneurons in the spinal cord (Rathelot and Strick, 2009). Recent work suggests that this rostral and caudal subdivision of the M1 hand area also exists in humans and maintains differences in function (Viganò et al., 2019). S1 has strong reciprocal connections with M1c, whereas PPC has comparatively weaker connections to M1r (Stepniewska et al., 1993). Lesions made independently to M1c and M1r in adult squirrel monkeys produced different deficits, where M1c lesions resulted in cutaneous sensory deficits, and M1r lesions produced errors in aiming of the hand (Friel et al., 2005). These results are not only consistent with the sensory inputs that are expected to arise from PPC and S1 but show the importance of sensorimotor integration such that different regions of M1 specialize in integrating the unique sensory information provided by PPC and S1. Furthermore, proprioceptive and visual inputs to input to M1 will be weighted differently depending on the goal of the task (Sober and Sabes, 2003) further attesting to the dynamic nature of sensorimotor integration in M1.

### M1 Plasticity and Sensorimotor Learning

In addition to the role of M1 in the production of movement, M1 also undergoes substantial plasticity, which has a critical role for learning skilled movements. Here, we define “motor learning” as an improvement in motor skill beyond baseline performance leading to a reduction in performance error that is retained over time (Shmuelof et al., 2012). Given that an error signal is inherently tied to sensory feedback and therefore needed for the learning of motor skills guided by sensory information (for review, see Seidler et al., 2013), we refer to motor learning as sensorimotor learning. Sensorimotor learning has been shown to induce functional and structural changes in M1 in rodents (Kleim et al., 1998) and non-human primates (Nudo et al., 1996a). In rodents, compared to practicing an unskilled lever-pressing task, practicing a skilled task that required specific paw manipulations to retrieve food pellets resulted in larger changes in M1 motor map representation of the forelimb, demonstrating that sensorimotor learning induces M1 plasticity (Kleim et al., 1998). M1 plasticity is defined as lasting changes in the morphological and/or functional properties of M1 (Sanes and Donoghue, 2000); experience-dependent plasticity is when these changes occur in response to life experiences, such as stroke (Kleim and Jones, 2008). In the rodent M1, plasticity underlying sensorimotor learning occurs through mechanisms of synaptic long-term potentiation (LTP) and long-term depression (LTD; Rioult-Pedotti et al., 2000). Similar to these results from rodent studies, the involvement of an LTP-like mechanism has been also demonstrated in plastic changes of M1 when adult humans practice ballistic thumb movements (Bütefisch et al., 2000). Importantly, in non-human primates, changes in M1 motor map representation of the distal forelimb were specific to skilled motor learning, whereas performing repetitive unskilled movements alone was not sufficient to induce changes in motor representations (Plautz et al., 2000). Additionally, disrupting M1 activity in humans with transcranial magnetic stimulation (TMS) immediately after motor practice can disrupt memory consolidation for that skill (Muellbacher et al., 2002b; Robertson, 2004) resulting in reduced learning, indicating the importance of M1 in the early consolidation of motor learning. The role of M1 plasticity in sensorimotor learning has also been demonstrated in the orofacial representations in humans (Arima et al., 2011) and nonhuman primates (Arce-McShane et al., 2014).

**Figure 3:**
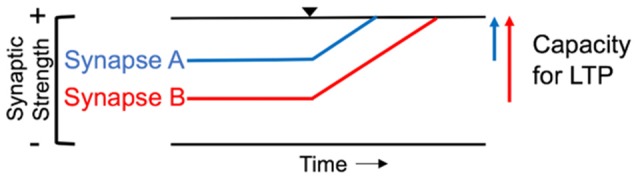
Homeostatic range of synaptic modifiability. In the illustration, *Synapse A* begins closer to its upper limit of modifiability (top black line) and has less capacity for long-term potentiation (LTP) than *Synapse B*. Black triangle denotes induction of LTP. *Synapse B* is further from the upper limit of synaptic strength, resulting in a greater capacity for LTP induction compared to *Synapse A*.

LTP in M1 is considered a primary synaptic process involved in the experience-dependent plasticity that underlies sensorimotor learning (Kleim et al., 1998; Bütefisch et al., 2000; Sanes and Donoghue, 2000; Ziemann et al., 2004; Nudo, 2013). At the synaptic level, a bidirectional range of dynamic modifiability exists, such that a synapse experiences a limited amount of synaptic strengthening (LTP) or reduction in strength (LTD; Rioult-Pedotti et al., 2000). The ability of a synapse to maintain a target range of modifiability to prevent over- or under-excitation of the neural circuit is referred to as homeostatic metaplasticity (Whitt et al., 2014). Evidence of synaptic metaplasticity suggests that prior history of synaptic plasticity influences the degree of future synaptic modification (Abraham and Bear, 1996). For instance, a synapse that is close to the upper limit of synaptic modifiability would not experience the same degree of LTP induction as a synapse farther away from its upper limit (Figure 3). Previous electrophysiological evidence from *in vitro* studies suggests that inducing LTD at a synapse, bringing it farther from its upper limit of modifiability, enhances the capacity for subsequent LTP induction (Rioult-Pedotti et al., 2000). This same principle has been demonstrated at the systems level (Ziemann et al., 2004). It was shown that sensorimotor learning reduced the capacity for subsequent LTP but enhanced the capacity for LTD in human M1. Additionally, the degree to which further LTP is blocked has been correlated with the magnitude of motor memory retention after sensorimotor learning (Cantarero et al., 2013a,b). Taken together, these results highlight the importance of experience-dependent plasticity in sensorimotor learning. LTP is largely mediated by glutamate, the primary excitatory neurotransmitter in the brain, and its interaction with the *N*-methyl-D-aspartate (NMDA) receptor throughout the cortex (Lüscher and Malenka, 2012). Functional inactivation of the NMDA receptor in M1 abolished the capacity for LTP induction *in vivo*, suggesting that these glutamatergic receptors are necessary for LTP to occur (Hasan et al., 2013). In addition to glutamatergic synapse contributions to experience-dependent plasticity, gamma-aminobuytric acid (GABA) synaptic modifiability is another important contributor to plasticity. GABA is the main inhibitory neurotransmitter in the brain (Blicher et al., 2015), and transient reductions in GABAergic inhibition have been shown to be necessary for LTP induction in M1 (Hess et al., 1996; Blicher et al., 2015; Kida et al., 2016).

In subsequent sections, we will review the importance of sensory inputs in shaping experience-dependent plasticity underlying sensorimotor learning under normal conditions and after stroke.

## The Role of Sensory Regions in Goal-Directed Hand Movements

### Posterior Parietal Cortex (PPC) as a Sensorimotor Integration Hub

The PPC is comprised of Brodmann Area (BA) 5, 7, 39 and 40 in the human brain and is anatomically connected to motor areas M1 and premotor cortex (PMC) *via* the superior longitudinal fasciculus (SLF; Makris et al., 2005; Koch et al., 2010). Although the PPC is not traditionally considered a primary part of the cortical motor network, it is involved in motor execution with populations of neurons that are motor dominant, in addition to populations that are visually dominant, or a combination of the two (Sakata et al., 1995). Non-human primate studies have demonstrated dense reciprocal PPC-M1 connections between the rostral strip of PPC and the medial lateral portion of M1 (Fang et al., 2005). Furthermore, regions of the PPC have distinct and direct pathways and networks with prefrontal motor cortical regions organized in functional zones (Gharbawie et al., 2011), which demonstrates the level of specific information the PPC can provide to the motor network. While PPC has been speculated to primarily influence M1 through polysynaptic connections with the PMC (Chao et al., 2015), support has been shown for monosynaptic projections from PPC to M1 (Karabanov et al., 2012). Additionally, in non-human primates, it has been shown that PPC has disynaptic connections with hand motoneurons in the dorsal horn and intermediate zone of the spinal cord (Rathelot et al., 2017), further suggesting potential contributions of PPC to the control of hand movements *via* the motor and sensory information PPC provides.

The PPC is a multisensory association area functioning to integrate different sensory modalities from visual, somatosensory, prefrontal and auditory inputs (Whitlock, 2017). The PPC has abundant reciprocal connections with sensory areas and is functionally parcellated such that the rostral portion of PPC is connected to somatosensory and motor regions, and the caudal portion of PPC has connections with visual and auditory regions (Stepniewska et al., 2009). The necessary inputs to PPC for sensorimotor processing needed for skilled hand movements include direct reciprocal inputs from the dorsomedial visual area that allows for continuous visual motion analysis necessary for interacting with the environment (Beck and Kaas, 1998; Kaskan and Kaas, 2007; Rosa et al., 2009; for review, see Kaas et al., 2011). Sensory inputs to BA 5 primarily come from somatosensory area S2 and the parietal ventral area, along with weaker inputs from S1 (Stepniewska et al., 2009). All three regions provide pertinent sensory information to PPC about proprioceptive and tactile activity of hand movements (Cohen et al., 1994; Prud’homme and Kalaska, 1994) that are important for sensorimotor integration used in hand exploration and object discrimination (Hinkley et al., 2007). Inputs to BA 5 are important as BA 5 is responsible for visuomotor transformations (Kalaska, 1996), making the PPC-M1 connection important for visuomotor control and visual-spatial processing (Binkofski et al., 1998; Mutha et al., 2011). PPC combines sensory signals about visual and kinematic reference frames into complex sensorimotor representations that are relayed to M1 to optimize motor commands (Sabes, 2011). PPC neurons are not only involved in control and error correction of a movement once initiated but are important for movement planning to achieve a motor goal (Mulliken et al., 2008; Aflalo et al., 2015), as neuronal firing also encodes movement intention (Snyder et al., 1997). Lesions in the rostral portion of PPC result in difficulty with shaping the fingers prior to grasping an object (Binkofski et al., 1998), further demonstrating an important role for PPC during the sensorimotor integration required for successfully performing goal-directed hand movements.

### Primary Somatosensory Cortex Involvement in Sensorimotor Integration

**Figure 4:**
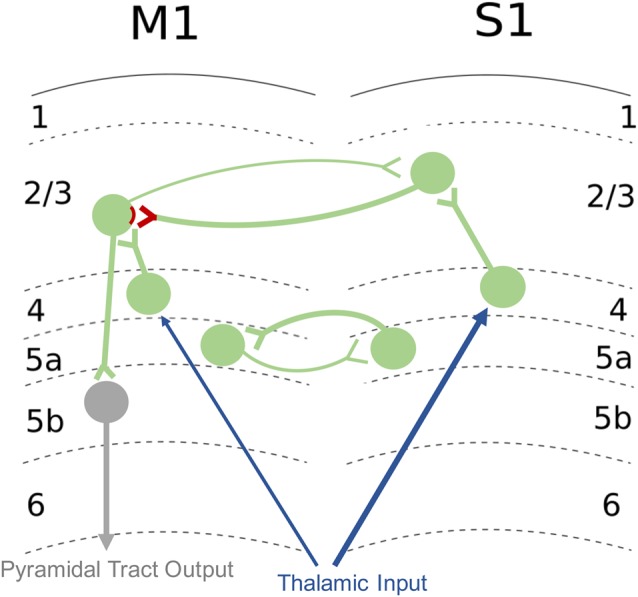
Excitatory M1-S1 connections. Sensory input from thalamus is relayed to layer 4 (L4) then to L2/3 of S1. S1 sends glutamatergic projections onto excitatory neurons in L2/3 of M1, and these synapses are sites of LTP and long-term depression (LTD) plasticity of connections involved in sensorimotor integration (denoted in red). Reciprocal connections of the M1-S1 loop are also shown. Pyramidal neurons from L2/3 of M1 project to output neurons in L5b of M1. Afferent inputs are shown in blue, intracortical connections are in green, and efferent outputs are shown in gray. Circles denote populations of neurons. Additional inputs and outputs are not shown. Refer to text for additional detail regarding M1-S1 connections.

In the human brain, S1 is comprised of BA 3a, 3b, 1, and 2 and receives direct somatosensory input from thalamus (Kaneko et al., 1994a). Somatosensory information is relayed from the periphery to the thalamus from the medial lemniscus (Boivie, 1978) *via* the spinothalamic tract (Boivie, 1979). Additionally, the posterior medial nucleus of the thalamus connects to inhibitory neurons in layer 1 (L1) of S1 that synapse onto the apical dendrites of neurons from other cortical layers (Castejon et al., 2016). Peripheral sensory information that is task-irrelevant can be filtered out through inhibition of afferent pathways *via* a process known as sensory gating (Eguibar et al., 1994). The thalamic relay nuclei are important for sensory gating, and lesions to the thalamus result in sensory gating impairments (Staines et al., 2002). This ascending sensory information can be modulated or gated by corticofugal descending projections from S1 to the dorsal column nuclei (Jabbur and Towe, 1961; Martinez-Lorenzana et al., 2001). Both S1 and M1 demonstrate somatotopic organization with representation of body regions localized to specific cortical cell columns (Kuehn et al., 2017). Furthermore, while M1 was previously thought to be agranular, it is now known that M1 shares the same structure as other primary cortical areas (Barbas and Garcia-Cabezas, 2015). The L4 in M1 is not cytoarchitecturally distinguishable, but electrophysiological studies have demonstrated it has traditional input/output proprieties: L4 receives excitatory input from the thalamus, has excitatory unidirectional outputs to L2/3, and weaker long-range corticortical connections (Yamawaki et al., 2014). However, there are distinct differences in that M1 has approximately half the amount of synapses that are exclusively excitatory whereas in S1, there are more synapses formed with both excitatory and inhibitory neurons (Bopp et al., 2017). It is proposed that M1 likely receives its feedforward inhibition through thalamacortical projections to L1 instead of L4 (Kuramoto et al., 2009; Bopp et al., 2017). In addition to connections from the thalamus, S1 also has direct projections to M1 that are important for the integration of somatosensory and motor information (Cash et al., 2015). In rodents, reciprocal projections connect the sensory representation in S1 to the corresponding motor representation in M1, creating a glutamatergic M1-S1 loop that connects L2/3 and 5a in S1 with L2/3 and 5a in M1 (Figure 4; Mao et al., 2011; Hooks et al., 2013). S1 relays somatosensory information through monosynaptic and polysynaptic connections to M1 (Kaneko et al., 1994a), and ongoing sensory input is used to refine and update descending motor commands (Rosenkranz and Rothwell, 2012). L2/3 neurons in M1 are able to directly excite pyramidal output neurons within the same cortical area (Kaneko et al., 1994b). At the network level, S1 activity has both excitatory (Rocco-Donovan et al., 2011) and inhibitory (Borich et al., 2015) effects on M1 at the network level. However, only excitatory projections from S1 to M1 have been characterized at the synaptic level (Papale and Hooks, 2018). The connectivity of inhibitory interneurons within M1 and how they are affected by sensory input have not been well studied. These S1-M1 connections provide an infrastructure for highly complex information integration that has the potential to be shaped and targeted for sensorimotor control and learning.

The ability of S1 to influence synaptic plasticity in M1 depends on sensorimotor synapses in L2/3 of M1. Synapses between S1 and M1 undergo plasticity that is driven by sensory input and results in the alteration of motor output (Kaneko et al., 1994a,b). These synapses are a main site of LTP and LTD in M1 (Kaneko et al., 1994a) and send excitatory projections to the pyramidal output neurons of M1 (Kaneko et al., 1994b; Huber et al., 2012). These connections allow for sensory feedback to shape motor output both in the short-term (immediate to minutes) and long-term (hours or longer). The ability for sensory input to influence motor output is specific to the connections between primary sensorimotor areas. Tetanic stimulation of S1, but not the ventrolateral nucleus of the thalamus, has been shown to produce LTP in L2/3 synapses of M1 (Iriki et al., 1989; Kaneko et al., 1994a). Tetanic stimulation of sensory thalamus only resulted in LTP in thalamocortical synapses with concurrent stimulation of S1 (Kaneko et al., 1994a). The S1-M1 connection has also been implicated in sensorimotor learning *in vivo* and is thought to be a main site of synaptic modifiability in response to motor skill learning (Papale and Hooks, 2018). These direct projections have been hypothesized to be a site of integration of sensory input and motor output and have an important role in guiding motor activity in response to sensory input (Hasan et al., 2013). One study in non-human primates demonstrated that ablation of S1 impaired the acquisition of motor skill but did not impair performance of the particular motor skill that had been learned previously, possibly due to intact thalamo-cortical connections that had been strengthened during skill training (Pavlides et al., 1993). Additionally, temporary inhibition of S1 in rodents has been shown to impair the ability to adapt motor performance based on changes in sensory input; however, basic motor patterns and motor commands that had learned previously were not affected (Mathis et al., 2017). Therefore, there is evidence to suggest that S1 is important for the ability to learn skilled movement and adjust motor plans to sensory input but may be less important for performance of overlearned or stereotyped movements in the upper limb. It should be noted, however, that ablation of other areas of S1, such as the face area, can lead to deficits in basic motor function, and previously learned motor tasks (Lin et al., 1993; Hiraba et al., 2000; Yao et al., 2002). In addition to connections between S1 and the ipsilateral M1, interhemispheric inhibitory connections between S1 s exist in humans (Ragert et al., 2011) and have been shown to influence plasticity in M1. For example, Conde et al. (2013) demonstrated that LTP-like plasticity in M1 induced by paired TMS and peripheral stimulation of the contralateral upper extremity switched to LTD-like plasticity when peripheral stimulation was applied to the upper limb ipsilateral to the TMS. These results demonstrate that the cortical sensorimotor circuitry that contributes to plasticity is not limited to one hemisphere, and interhemispheric network connectivity likely influences sensorimotor learning. However, the specific involvement of S1 in motor performance will depend on the characteristics of the task including the importance of sensory information for skilled performance.

## Impact of Stroke on Sensorimotor Integration and Learning

### Sensorimotor Deficits After Stroke

The impact of stroke on sensorimotor integration depends on the location of the stroke. Because the MCA supplies both the motor and sensory regions and is the most common type of stroke (Walcott et al., 2014), stroke in this vascular territory has a great likelihood of affecting sensorimotor integration. Therefore, our discussion is primarily focused on MCA strokes affecting the sensorimotor cortex although strokes in other vascular territories may also impact sensorimotor integration (Staines et al., 2002). There are dynamic processes post-stroke that change as a function of time and affect the neurophysiology of sensorimotor integration. Time post-stroke is defined in phases: hyper-acute (0–24 h); acute (1–7 days); early subacute (7 days–3 months); late subacute (3–6 months); and chronic (>6 months; Bernhardt et al., 2017b). Initial neuronal cell death in the lesion core leads to both structural and functional disconnection with brain regions outside the primary area of infarct (Carrera and Tononi, 2014). Motor recovery occurs in part from spontaneous biological repair (SBR) that transitions from a state of cell death and inflammation to a state of increased neuronal excitability and experience-dependent plasticity lasting ~3 months post-stroke (Cramer, 2008). Most recovery post-stroke occurs rapidly in the early sub-acute phase and the magnitude of improvement slows down in the late sub-acute phase (Lee et al., 2015). In the chronic phase post-stroke, patients have reached a stable, though modifiable plateau in motor recovery (Jørgensen et al., 1995) with less than 20% of patients experiencing full recovery of upper extremity motor function (Kwakkel et al., 2003).

Upper extremity paresis is the most predominant motor impairment after MCA stroke, which results from a lesion involving the CST that is also necessary for skilled hand movements (Lang and Schieber, 2003). Paresis can contribute to deficits in both the initiation and termination of voluntary movement of the wrist (Chae et al., 2002). Other motor deficits include spasticity and impaired motor control (Raghavan, 2015), with 85% of patients in the chronic phase post-stroke still possessing residual motor deficits (Lee et al., 2015).

Common somatosensory modalities affected after stroke are tactile sensation, proprioception, and stereognosis (Connell et al., 2008). It has been recently reported that 62% of acute stroke patients demonstrated deficits in their ability to locate their hand and arm in space (Findlater et al., 2016). Deficits in proprioception have direct implications as information about the arm and hand are necessary for proper movement and important for improving sensorimotor function after stroke (Aman et al., 2014). Due to the reliance of the motor system on sensory information for movement optimization, sensory impairment is expected to have motor repercussions. Similarly, sensory deficits can occur even when there are ischemic lesions specifically in the M1 motor pathway and not in somatosensory afferents (Nudo et al., 2000), suggesting that sensory integration can be disrupted even in the absence of a lesion present in sensory afferent pathways. Clinically, sensorimotor deficits are usually discussed in terms of sensory deficits and motor deficits assessed separately. Sensory and/or motor deficits after stroke have been routinely measured using observer-based clinical scales either focused on measuring level of impairment, with scales such as the Fugl-Meyer Assessment (Fugl-Meyer et al., 1975) and Nottingham Sensory Assessment (Lincoln et al., 1998), or focused on measuring level of function with the Wolf Motor Function Test (Wolf et al., 2001) and the Jebsen Taylor Hand Test (Jebsen et al., 1969). However, there are several limitations of standard observer-based clinical assessments including: decreased reliability and sensitivity compared to objective assessments, lack of precision with non-continuous data, and greater susceptibility to floor and ceiling effects of performance (Scott and Dukelow, 2011). Therefore, there is a need for objective assessments to better characterize post-stroke sensorimotor deficits.

### Assessment of Sensorimotor Integration After Stroke

In addition to the need for objective assessments of sensorimotor deficits, it is important to examine the impact of stroke on sensorimotor integration to better understand the relationship between sensory and motor deficits. As defined earlier, sensorimotor integration is the ability to incorporate sensory inputs to shape motor output (Wolpert et al., 1998). Therefore, examining the effects of manipulating sensory information on motor output can be employed to evaluate sensorimotor integration. For a detailed review of various measurement techniques, see Riemann et al. (2002). One approach utilizes robotic-based technologies during visually guided upper extremity tasks to quantify aspects of sensorimotor control. Coderre et al. (2010) examined the characteristics of feed-forward control and feedback control of stroke patients in the early sub-acute recovery period. It was observed that most patients with deficits initiating movement also had deficits with adjusting movement from sensory feedback, emphasizing that movement difficulties were not solely due to motor impairments but also due to an inefficiency with integration of sensory modalities. Using another robotic-based assessment, it was shown that kinesthetic impairments post-stroke were not resolved with the addition of visual information indicating the location of the arm in space (Semrau et al., 2018). This observation was unique to stroke patients in comparison to healthy controls and the impairment was attributed, in part, to damage to the PPC. Studies have previously shown that sensorimotor abnormalities during motor control are related to parietal lesions (Desmurget et al., 1999; Findlater et al., 2016), signifying the important role of PPC in sensorimotor integration.

In addition to robotic-based assessments, sensorimotor integration has also been probed using non-invasive stimulation in humans. Combining a peripheral sensory stimulus with non-invasive brain stimulation using TMS can measure the effects of afferent sensory input on the magnitude of TMS-evoked motor output. One example assessment is short-latency afferent inhibition (SAI) where somatosensory input from peripheral stimulation of the median nerve can inhibit motor output to hand muscles (Tokimura et al., 2000). In the acute phase post-stroke, patients have reduced SAI compared to healthy controls (Di Lazzaro et al., 2012), that seems to normalize in the chronic phase where there is no significant difference in SAI between patients and controls (Brown et al., 2018). Sensorimotor integration may be disrupted shortly after stroke but this reduction was correlated with improved outcome in the chronic phase (Di Lazzaro et al., 2012) This suggests that while decreased SAI may be beneficial acutely, it must normalize chronically for improved motor function. Previous work has also shown that the integration of S1 afferent input to M1 decreased acutely but was more comparable to healthy controls at 6 months post-stroke; this finding also paralleled improvement in sensation (Bannister et al., 2015). Sensorimotor integration has also been assessed using a vibration-based sensory stimulus of the muscle belly preceding TMS. It was found sensorimotor integration was abnormal in chronic stroke patients and greater abnormality was associated with greater magnitude of motor impairment and dysfunction (Brown et al., 2018). Taken together, these studies demonstrate that sensorimotor integration is impacted differentially depending on time post-stroke and the type of sensory information provided, but overall is an important process during recovery.

### Plasticity and Sensorimotor Learning After Stroke

Many cellular and synaptic processes contribute to plasticity after stroke. In the acute phase after stroke, LTP is facilitated in the perilesional areas, suggesting an amplification of network plasticity that influences cortical reorganization (Hagemann et al., 1998). Neuroplasticity is enhanced through processes such as axonal sprouting and GABA receptor downregulation (Carmichael, 2016). Additionally, functional recovery is most rapid during this early time period, occurring in the first 3 months for humans and roughly 1 month for rodents (Caleo, 2015). Plasticity subsequently plateaus in the chronic stage of recovery (Hendricks et al., 2002; Hara, 2015). Rehabilitative interventions have been shown to be most effective when initiated early after stroke and become less effective with time post-stroke (Biernaskie et al., 2004). Despite the plateau in neuroplasticity during the chronic phase of recovery, it is currently unclear whether this level differs from that of matched healthy controls. In a study by Zeiler et al. (2016) in a rodent model of chronic stroke, the induction of a second stroke enhanced plasticity and response to skilled motor training, indicating that it is possible to reopen this window of enhanced plasticity during the later stages of recovery. Increasing the capacity for neuroplasticity during the chronic stage of recovery has the potential to enhance recovery of function for stroke survivors with persistent motor-related disability.

As mentioned previously, GABAergic activity is strongly related to synaptic plasticity in healthy individuals. In rodent models of cerebral ischemia, GABAergic inhibition has been shown to be elevated within minutes (Globus et al., 1991), a potentially neuroprotective mechanism to counteract excitotoxicity caused by excess glutamate release (Pellegrini-Giampietro, 2003). GABA levels return to baseline within an hour of reoxygenation (Schwartz-Bloom and Sah, 2001). Reductions in GABAergic inhibition continue during the acute phase after stroke, and this process has been related to functional motor recovery in mice (Clarkson et al., 2010). It has been suggested that this reduction in GABAergic activity serves to facilitate neuroplasticity in M1 through unmasking of existing, inactive synaptic connections (Paik and Yang, 2014), the development of new connections (Murphy and Corbett, 2009), or the induction of LTP (Hess et al., 1996; Sanes and Donoghue, 2000). While GABAergic activity has been shown to be an important contributor to plasticity after stroke, other mechanisms, such as brain derived neurotrophic factor (BDNF) and neuromodulin signaling, have been implicated as well. For in-depth reviews of cellular and synaptic mechanisms of plasticity after stroke, see Murphy and Corbett (2009) and Alia et al. (2017). Given that similar mechanisms are thought to underlie neuroplasticity and functional recovery after stroke (Kleim and Jones, 2008), therapeutic strategies that optimally promote neuroplasticity hold promise for improving the rate and magnitude of functional recovery after stroke.

As discussed earlier, motor skill learning has been shown to induce structural and functional changes in M1 that underpin sensorimotor learning in rodents (Kleim et al., 1998), non-human primates (Nudo, 2013), and healthy humans (Bütefisch et al., 2000; Sanes and Donoghue, 2000; Ziemann et al., 2004). It has also been shown that motor skill learning underlies recovery of function after stroke in humans (Krakauer, 2006) and non-human primates (Nudo et al., 1996b). One mechanism underlying recovery is the preservation or expansion of the M1 representation of the affected hand. Skilled motor training after stroke in non-human primates prevented the reduction of the affected distal upper extremity representation in M1 that occurred after an equivalent period of no training (Nudo et al., 1996b). In some cases, the hand representation expanded into representations for adjacent body parts after training, and this reorganization of M1 corresponded to better recovery of skilled hand function. It has also been shown that S1 activity contributes to sensorimotor learning and recovery after stroke in humans and non-human primates. Nudo et al. (2000) demonstrated that impairments in sensory inputs to M1 after stroke in non-human primates contributed to motor deficits in a task that required skilled hand movements. In humans, continuous theta burst stimulation (cTBS), a TMS paradigm that can decrease excitability of the stimulated area, delivered over contralesional S1 in order to reduce transcallosal inhibition on ipsilesional S1 was shown to enhance motor recovery after stroke (Meehan et al., 2011). Another study by Brodie et al. (2014) demonstrated that excitatory rTMS to the ipsilesional S1 paired with motor skill training increased sensorimotor learning compared to stimulation or skill training in isolation. Therefore, attempting to enhance S1 excitability and/or sensorimotor integration may offer an effective approach to improve sensorimotor learning and functional recovery after stroke.

## Strategies to Modulate Sensorimotor Integration and Potential Therapeutic Effects After Stroke

### Current Therapeutic Interventions

Sensorimotor integration occurs across the neuraxis and therefore provides multiple potential targets for therapeutic intervention. Several experimental procedures have been developed to modulate afferent input to M1, and therefore sensorimotor integration, in humans. Peripheral vibration is a neuromodulation approach that increases afferent input that is thought to modulate M1 excitability by regulating the activity of cortical inhibitory interneurons that are involved in motor output (Rosenkranz and Rothwell, 2006). This increase in afferent input is thought to change the response of M1 to sensory input and therefore influence sensorimotor integration in the cortex. Both focal (Celletti et al., 2017) and whole-body vibration (Boo et al., 2016) have shown promise in improving upper extremity function in individuals with stroke. However, across studies, the effectiveness of vibration to improve post-stroke motor function remains unclear (Liao et al., 2014; Park et al., 2018).

In contrast to increasing afferent input to M1, models of temporary deafferentation have shown promise in targeting sensorimotor integration by reducing sensory input to modulate motor output. In rodents, transection of the facial nerve leads to a rapid expansion of the adjacent forelimb representation in M1, likely due to rapid removal of GABAergic inhibition (Sanes et al., 1988; Huntley, 1997). This concept has been applied non-invasively in humans by temporarily reducing afferent input from a portion of the upper extremity to M1 with the goal of reducing GABAergic inhibition to adjacent areas of the limb. It is thought that rapid unmasking of horizontal connections leads to an expansion of the cortical representation. Targeting this mechanism, several temporary deafferentation strategies have been studied in humans with the goal of increasing M1 representation of the affected limb to improve functional outcomes after stroke. Ischemic nerve block (INB) of the arm is one method that serves as a model of transient segmental deafferentation in humans. Using a pneumatic tourniquet at the elbow, afferent sensory inputs from the distal forearm to the sensorimotor cortex are restricted, leading to an increase in excitability of cortical representations of muscles immediately proximal to the deafferented forearm (Brasil-Neto et al., 1993). However, this form of INB may be less applicable for individuals with stroke, as a main goal of stroke rehabilitation is to improve hand function, and it appears that INB effects more proximal parts of the arm (Lang et al., 2013). A different approach that has been shown to increase motor function after stroke is the application of anesthesia to areas proximal to the hand, such as the brachial plexus (Muellbacher et al., 2002a) or forearm (Sens et al., 2012, 2013), simulating deafferentation of the upper or lower arm, respectively. After applying anesthesia to the brachial plexus of the affected arm, Muellbacher et al. (2002a) demonstrated an improvement in motor skill after training in individuals with chronic stroke compared to training without anesthesia. Additionally, there was an increase in motor output in response to TMS application with no change in motor threshold, suggesting a rapid cortical reorganization and reduction in inhibition. Application of anesthetic cream to the forearm, another region proximal to the hand, improved somatosensory and motor function distal to the site of application in individuals with chronic stroke (Sens et al., 2013). Blood flow restriction (BFR) is another technique that uses a pneumatic cuff applied to the arm to reduce blood flow to a target level that is maintained during exercise (Yasuda et al., 2014). Brandner et al. (2015) showed that BFR during resistance exercise increases corticomotor excitability, and this effect is thought to be mediated by the reduction in cortical afferent input. A primary concern for the use of INB and BFR in a rehabilitation setting is that the use of a tourniquet or arm cuff poses a risk for individuals with sensory impairments and/or cardiovascular irregularities, such as individuals with stroke (Spranger et al., 2015). Therefore, individuals with stroke may benefit from a method of temporary deafferentation with fewer potential risks.

### Future Directions for Therapeutic Interventions

Short-term immobilization of the arm is a safe, low-cost approach for the transient modulation of sensorimotor cortical function in healthy individuals. In humans and animals, prolonged immobilization or disuse of a limb can occur after neurological insult that induces maladaptive plasticity, such as reduction in cortical representations of the limb (Pons et al., 1991; Langer et al., 2012; Milliken et al., 2013; Viaro et al., 2014), which can contribute to “learned nonuse” and a compensatory reliance on the unaffected limb (Wolf, 2007). While learned nonuse and its effects on cortical organization have been examined, short-term immobilization has been less well-studied. Short-term arm immobilization (typically 8 h) reduces sensory input to, and motor output from, the contralateral sensorimotor cortex resulting in transiently decreased M1 and S1 cortical excitability following immobilization in healthy individuals (Huber et al., 2006; Rosenkranz et al., 2014). This decrease in excitability is thought to be driven by LTD-like processes (Huber et al., 2006). Allen et al. (2003) demonstrated that whisker deprivation in rodents induced LTD-like effects in sensorimotor areas that occluded further LTD induction but enhanced LTP induction in slice preparations, consistent with the model of homeostatic metaplasticity. Short-term immobilization of the arm has been proposed as a strategy to induce LTD-like plasticity and enhance the capacity for LTP induction in the human motor cortex. Indeed, a single short bout (8 h) of immobilization temporarily reduced TMS-based measures of cortical excitability; however, the capacity for synaptic strengthening was significantly enhanced (Rosenkranz et al., 2014). However, the behavioral effects of this enhanced synaptic strengthening are currently unclear.

Given that short-term immobilization modulates excitability of S1 and M1, it is likely that immobilization impacts the integration of sensory and motor information that underlies experience-dependent plasticity. Therefore, short-term immobilization could potentially modulate neural processes underlying sensorimotor learning. However, the effects of immobilization on sensorimotor learning have not been well studied in humans. To our knowledge, only one study has examined sensorimotor learning after short-term arm immobilization (Opie et al., 2016) and did not show a clear effect of immobilization on sensorimotor learning. The lack of effect could be due, in part, to the high number of individuals with the BDNF Val66Met polymorphism that is associated with reduced use-dependent plasticity in sensorimotor areas (Kleim et al., 2006). Given the relationship between neural plasticity and sensorimotor learning, further examination of the effect of short-term arm immobilization on sensorimotor learning is warranted. Short-term arm immobilization could show promise as a rehabilitative intervention to increase post-stroke sensorimotor recovery by enhancing the capacity for neuroplasticity leading to better training-related increases in motor function. More broadly, given its demonstrated role in motor control, promotion of sensorimotor integration plasticity has potential as a therapeutic strategy post-stroke.

## Conclusion

Skilled hand movements are necessary for normal function in daily life but are frequently impaired after stroke. Goal-directed functional movements rely on accurate integration sensory information and when sensorimotor integration is compromised, movement ability is compromised. Despite the importance of sensory contributions to normal and abnormal movement, research has predominantly focused on motor aspects of stroke recovery. Given that sensorimotor integration has been shown to be negatively impacted after stroke and correlated with level of recovery, there is an increasing need to focus future research efforts towards comprehensive characterization of the neural mechanisms of sensorimotor integration and their contributions to functional movements in both health and disease. Furthermore, an increased understanding of contributions of sensorimotor integration and sensorimotor learning to skilled hand movements post-stroke will likely offer new rehabilitative targets to increase the recovery of function after stroke.

## Author Contributions

LE and EK planned, drafted, and edited the manuscript. CB and MB planned and edited the manuscript.

## Conflict of Interest Statement

The authors declare that the research was conducted in the absence of any commercial or financial relationships that could be construed as a potential conflict of interest.
